# Developing the questionnaire of general population knowledge, attitudes and practices towards the COVID‐19 outbreak

**DOI:** 10.1002/nop2.2143

**Published:** 2024-03-23

**Authors:** Hossein Namdar Areshtanab, Maryam Vahidi, Mina Hosseinzadeh, Zahra Khani

**Affiliations:** ^1^ Department of Psychiatric Nursing, Nursing and Midwifery Faculty, Social Determinants of Health Research Center Tabriz University of Medical Science Tabriz Iran; ^2^ Social Determinants of Health Research Center Tabriz University of Medical Sciences Tabriz Iran; ^3^ Department of Community Health Nursing, Nursing and Midwifery Faculty Tabriz University of Medical Sciences Tabriz Iran; ^4^ Tabriz University of Medical Science Tabriz Iran

**Keywords:** attitude, COVID‐19, knowledge, nurses, public health

## Abstract

**Aim:**

To develop the general population knowledge, attitudes and practices (KAP) questionnaire towards the COVID‐19 outbreak.

**Design:**

A methodological study.

**Methods:**

The general population KAP questionnaire items were designed using a literature review. A panel of experts was used to calculate content validity ratio (CVR) and content validity index (CVI). Construct validity was examined using exploratory factor analysis (EFA) and hypothesis testing. Internal consistency of the questionnaire was measured using Cronbach's *α* coefficient. Eight hundred forty‐seven patients referred to health centres in Tabriz, Iran completed the questionnaire in 2021.

**Results:**

The developed questionnaire consisted of three parts, including knowledge, attitudes and practices. EFA identified three dimensions for the knowledge (ways of transmission and prevention, high‐risk groups, and symptoms and treatment), three dimensions for the attitudes (hope, fear and view of the vaccine) and three for the practices (adherence to personal and public hygiene, limiting their presence in society and protecting yourself in the community). The Kaiser–Meyer–Olkin index for knowledge, attitudes and practices was 0.733, 0.725 and 0.886, respectively, with a significant Bartlett's test of sphericity (*p* < 0.01). The hypotheses of knowledge and attitudes that are the related factors of practices were confirmed. Cronbach's *α* was 0.63, 0.74, 0.77, 0.1 and 0.85 for knowledge; fear, hope and view of the vaccine subscales of attitudes; and practices, respectively.

**Patient or Public Contribution:**

The developed 47‐item questionnaire had acceptable validity and reliability. Thus, nurses can use it to assess the clients' KAP during the COVID‐19 outbreak. Also, nursing researchers can use this questionnaire in their descriptive and interventional studies.

## 1 INTRODUCTION

With the rapid increase in cases of COVID‐19 disease reported worldwide, the World Health Organization (WHO) declared the outbreak a global pandemic and called on all countries to work together to deal with the disease (Tang et al., 2020). Regardless of the countrywide measures in fighting the outbreak, the achievement or failure of these efforts depends on public conduct. In particular, public adherence to preventive measures, encouraged through the public's knowledge and attitudes towards COVID‐19, is essential to prevent the spread of the disease. Evidence shows that public awareness is critical in dealing with the pandemic (Al‐Hanawi et al., 2020). Basic knowledge of the outbreak helps understand the risk behaviours and allows the general public to respond quickly to the pandemic. Research on population knowledge, attitudes and behaviours can guide preventive strategies throughout the event and inform planning for future outbreak preparedness (Johnson & Hariharan, 2017).

The knowledge, attitudes and practices (KAP) theory divides the changes in human behaviour into three steps, including knowledge acquisition, attitudes generation and behaviour formation. This theory states that a relationship exists between knowledge, attitudes and behaviours, so knowledge is the basis of behaviour change and attitude is the driving force. Furthermore, based on the health belief model, the formation of health beliefs plays an essential role in accepting and changing lousy behaviour and adopting healthy behaviour (Fan et al., 2018).

Accordingly, analysing and defining the concepts of KAP towards COVID‐19 is crucial for nursing researchers, providing them with a common understanding of the topic and areas that can be developed. Then, they can design instruments to measure it, determine the factors affecting its improvement and plan appropriate interventions. Therefore, the results can be applied to nursing practice, research and policy. For example, the designed questionnaire can be used as a guide for patient evaluation and data collection in the assessment phase of the nursing process. They can also examine the effectiveness of interventions in improving the clients' knowledge, attitudes and practices. The results can contribute as a study instrument to help researchers and managers regarding the general public's knowledge, attitudes and practices in performing research and designing policies.

So far, studies examining the individuals' KAP towards COVID‐19 have used questionnaires whose psychometric properties have yet to be assessed except in two studies (Park, 2021; Saefi et al., 2020). The questionnaire designed by Saefi evaluates undergraduate students' KAP to prevent COVID‐19 infections. Park's questionnaire of KAP regarding COVID‐19 was designed and validated for the target group of nursing students. Students are an educated group with a different lifestyle and health literacy from the general population. Therefore, this study aimed to develop a questionnaire of the general population KAP towards COVID‐19 and psychometrically measure it.

## 2 METHODS

**FIGURE 1:**
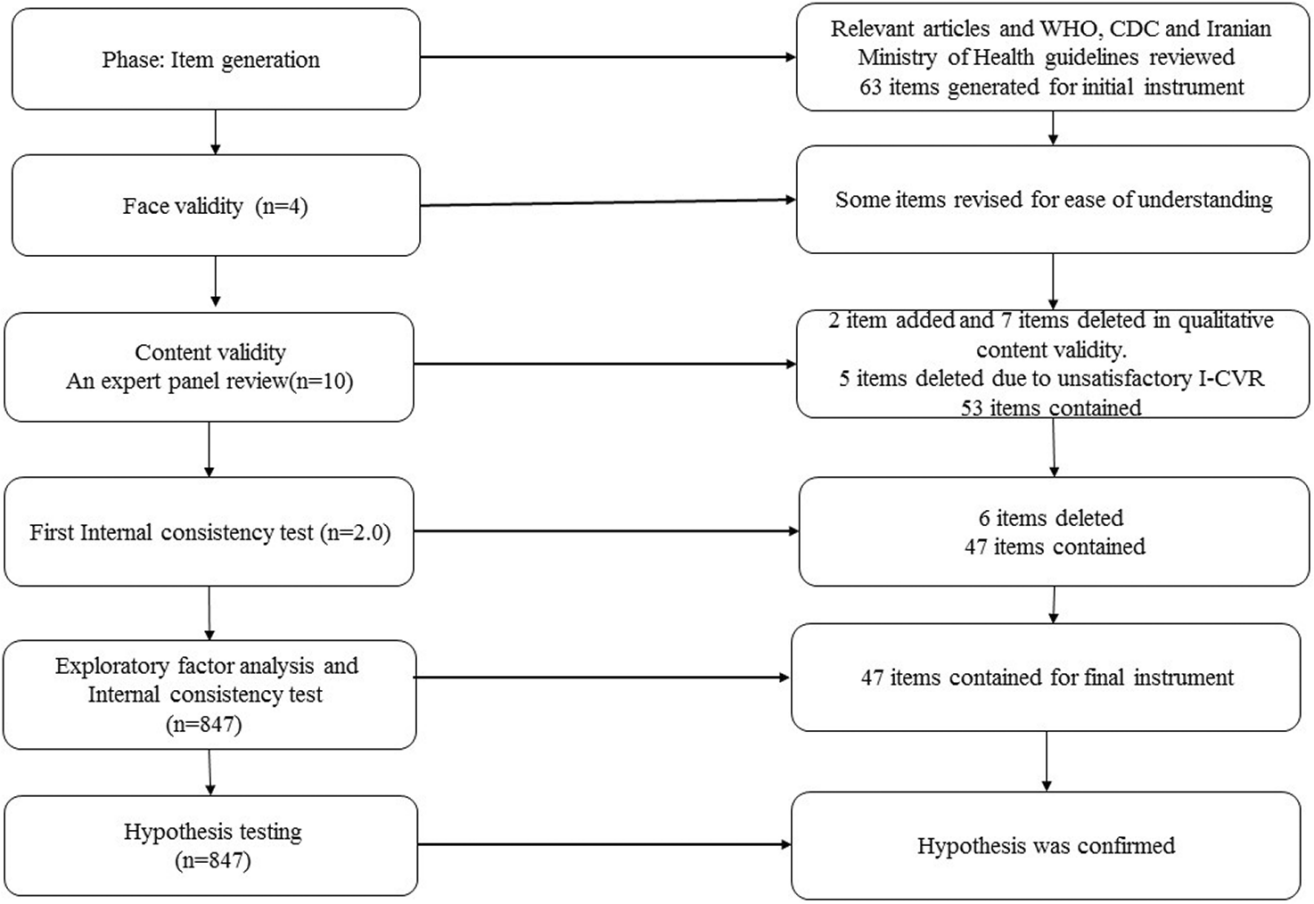
A flow diagram of the development and validation process.

This methodological study implemented three main stages to design the questionnaire. Items were initially generated with an extensive literature review, followed by checking face validity, content validity and internal consistency. Finally, construct validity and final internal consistency were checked (Figure 1).

We searched the databases, including Sage, Scopus, PubMed, IranDoc, Magiran, SID and MEDLIB, using keywords such as coronavirus, COVID‐19, knowledge, attitudes and practice, to extract the questions used in the related articles (Al‐Hanawi et al., 2020; Moradzadeh et al., 2020; Sakr et al., 2021; Xu et al., 2021; Zhong et al., 2020). The guidelines of the WHO, Centers for Disease Control and Prevention (CDC) and the Iranian Ministry of Health were also reviewed. The questionnaire items of the papers were studied to remove all duplicate and overlapping items, resulting in a total of 63 items for the initial questionnaire.

Four clients referred to one health centre in Tabriz were interviewed face‐to‐face to examine the questionnaire's difficulty level, appropriateness and ambiguity (face validity). Ten experts suggested editing, merging, deleting or adding items to determine qualitative content validity. The content validity ratio (CVR) and content validity index (CVI) were used for quantitative content validity. According to Lawshe's table, the cut‐off point of 0.62 was considered for CVR, and the Kappa statistic was used with the cut‐off of 0.8 to determine the CVI. Therefore, the CVI values for each item and the full scale (the average calculation approach) were determined. Finally, the questionnaire was prepared for the quantitative part.

The participants of the quantitative part included 847 clients referring to health centres affiliated with Tabriz University of Medical Sciences, Tabriz, Iran. The acceptable sample size for such studies is 2–20 participants per item (Anthoine et al., 2014); therefore, according to the number of questionnaire items, 850 individuals were considered as the sample size. The inclusion criteria were age over 18 years, not suffering from cognitive disorders such as dementia or delirium according to the participants' statement or their health record and having the physical ability to answer the questions.

A simple sampling method was used to select 10 health centres (of 83) in Tabriz, to which the researchers referred from August to October 2021 and took samples using the convenience sampling method. The questionnaire was distributed among 20 people in the first stage to determine internal consistency. Also, the items were analysed with the loop method and the correlation coefficient of the items with the whole questionnaire (corrected item–total correlation coefficients [CITC]), removing items that had negative or close to zero coefficients. The loop analysis method determined the items whose removal significantly changed the *α* coefficient. An electronic version of the questionnaire was used to prevent the spread of coronavirus infection. The primary researcher read the questionnaire items to participants and recorded data on a laptop device. All questionnaires were completed in a private room to protect the privacy of responses. Data were analysed using SPSS v.11.5 (SPSS11.5, Inc, USA).

The construct validity of the instrument was evaluated using EFA and hypothesis testing. Before conducting the EFA, the common variance of each item was examined with other items using the principal axis factoring method. Items that had a common variance of <0.3 were excluded, after which the Kaiser–Meyer–Olkin (KMO) and Bartlett's test of sphericity (BT) were also performed. The factors were extracted after preparing the correlation matrix, with the principal axis factoring analysis method, the eigenvalues and variance corresponding to the factors before and after direct oblimin rotation and using the Scree plot chart. Items with factor loading of <0.3 were removed. Cronbach's *α* coefficient was calculated after selecting the final items of the scale based on item and factor analysis. Besides, the usability of the questionnaire was also examined.

In addition to EFA, “hypothesis testing” was used according to COSMIN's guide to check the construct validity of the questionnaire (Mokkink et al., 2010). Before data collection, it was assumed that improving people's knowledge and attitude was associated with enhancing their practice when the effect of other variables is controlled. Therefore, practice was selected as the outcome variable. Independent variables, including age, gender, number of children, history of COVID‐19 infection, history of COVID‐19 infection in family or friends, education, the priority of the source of information about COVID‐19, job, marital status and subscales of attitudes and knowledge, were entered into a multiple linear regression model with the enter method. The independent variables in this study were a mixture of continuous and categorical variables. In multiple regression analyses, the categorical variables with more than two groups were coded as “dummy variables” (Kleinbaum et al., 2013). All assumptions of linear regression analysis (linearity, normality and independence of error terms, as well as multicollinearity of independent variables using the variance inflation factor of tolerance) were examined. A *p*‐value of 0.05 (two‐sided) was used to denote statistical significance. The regression coefficient and 95% confidence intervals were reported to consider the strength of the association.

### 2.1 Ethical consideration

The research was approved by the Ethics Committee of Tabriz University of Medical Sciences (ethical code: IR.TBZMED.REC.1399.985). Participants were informed regarding the research goals, voluntary participation, confidentiality and anonymity of data, and the right to leave the research at any desired time, and then, written informed consent was obtained.

## 3 RESULTS

After reviewing the texts and examining various instruments and guidelines, the initial draft was presented in three sections: knowledge, attitudes and practices. The knowledge section included 24 items about transmission methods, clinical symptoms, treatment, risk groups, isolation, prevention and control. The respondents chose one of the following answers: correct, incorrect and I do not know. The second section had 13 items and assessed the participants' attitudes towards COVID‐19 using a five‐point Likert scale (strongly disagree, disagree, neutral, agree and strongly agree). The final section of the questionnaire, with 26 (yes/no answer) items, evaluated the respondents' practices during the COVID‐19 pandemic.

In assessing face validity and qualitative content, the ambiguities raised were resolved according to the opinion of the participants and experts. In the qualitative content validity, two items were added, three items were deleted in the knowledge section, and four items were deleted in the practice section. The CVR of two items in the knowledge section and three in the practice section were less than the cut‐off point, leading to their removal after consulting with the research team. No items were deleted or added in the attitudes section. In assessing CVI, the Kappa statistic was not less than 0.8 in any items, so none were removed. In the second round, the questionnaire was provided to six experts, indicating values of 0.92, 0.89 and 0.95 for the S‐CVI of the knowledge, attitude and practice sections, respectively.

### 3.1 Internal consistency of the first stage

Cronbach's *α* was 0.484, 0.72 and 0.80 for knowledge, attitude and practices, respectively. Five items in the knowledge section and one in the practice section were removed due to their low correlation coefficient with the whole instrument. In this way, Cronbach's *α* coefficients for knowledge and practices increased to 0.63 and 0.87, respectively. According to the analysis results using the loop method, removing none of the items significantly changed the *α* coefficient of the total knowledge, attitudes and practices. Therefore, none of the items were removed by this method.

**FIGURE 2:**
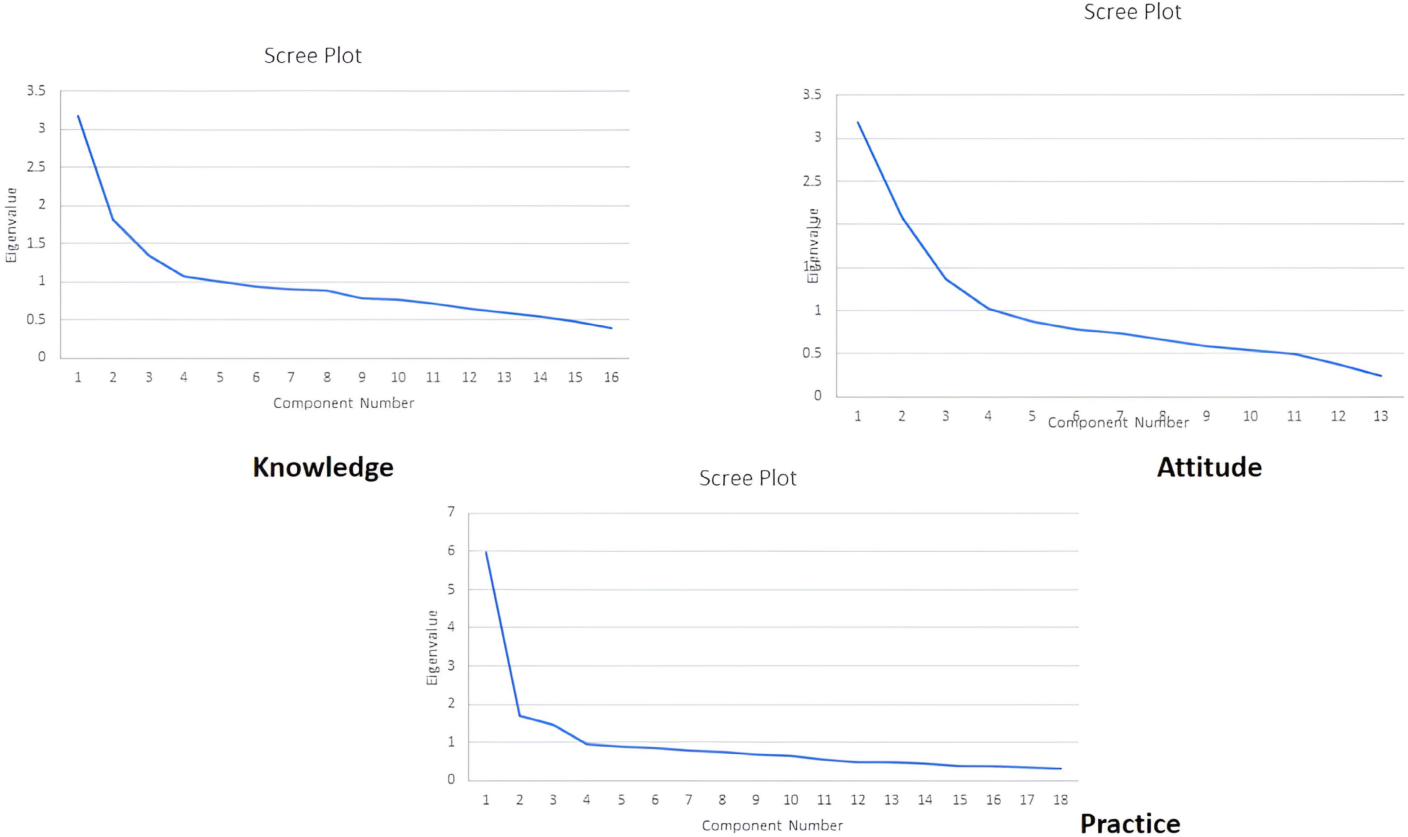
Scree plot of knowledge, attitudes and practices.

The current study analysed 847 questionnaires. The participants' mean (SD) age was 41.27 (15.24) years. Of 26% had no children, 52% had one to three, and the rest had four to nine children. Other personal and social characteristics of the participants are listed in Table 1. The KMO index for knowledge, attitude and practices sections was 0.733, 0.725 and 0.886, respectively. Bartlett's sphericity test was also significant (*p* < 0.01, df = 120 and *χ* ^2^ = 1894.933 for the knowledge section, *p* < 0.01, df = 78 and *χ* ^2^ = 2472.424 for the attitude section, and *p* < 0.01 df = 153 and *χ* ^2^ = 4954.420 for practice section), revealing detectable relationships between scale items. The mentioned items were subjected to factor analysis. The five extracted factors of knowledge, the four initial extracted factors of attitude and the three initial extracted factors of practices explained 51.66%, 58.92% and 50.62% of the initial variance before rotation, respectively. Three primary factors in each section were selected based on the Scree plot diagram (Figure 2). It is worth noting that the selected factors of the knowledge, attitude and practices sections explained 38.50%, 50.99% and 50.62% of the variance, respectively.

**TABLE 1:** Demographic features of participants.

Variables	*N* (%)
Gender	
Male	246 (29)
Female	595 (70.2)
Education	
Diploma and lower	517 (61)
Undergraduate	233 (27.5)
Master	70 (8.3)
PhD	18 (2.1)
Marital status	
Single	268 (31.7)
Married	575 (67.9)
Divorced	16 (1.9)
Job	
Unemployed	16 (1.9)
Housewife	74 (8.7)
University student	128 (15.1)
Worker	146 (17.2)
Employee	3 (0.4)
Self‐employed	27 (3.2)
Retired	39 (4.6)
Not reported	414 (48.9)
History of COVID‐19	
Yes	298 (35.2)
No	545 (64.3)
History of COVID‐19 in family and friends	
Yes	508 (60)
No	331 (39.1)

Table 2 shows the results of the EFA. The researchers checked the conceptual relevance of each item with other items of the factors. Therefore, some items were moved among the factors. In the knowledge section, item 6 was transferred from the first factor and item 5 from the third factor to the second factor. Item 10 was transferred from factor two and items 1 and 2 from factor three to factor one. In the practice section, item 15 was transferred from the first to the second factor and item 18 from the second to the first factor. Also, item 16 was transferred from the first to the third factor. In the attitude section, two items had cross‐loadings. In one factor, the item with a larger load was considered, and in the other, the item with semantic proximity was considered. Item 11 was transferred from the third to the first factor. Then, the factors were named.

**TABLE 2:** Scale item loadings for three sections of questionnaire.

Knowledge				Attitudes				Practices			
Component				Component				Component			
	1	2	3		1	2	3		1	2	3
K15	0.613	–	–	A7	0.736	–	–	P13	0.739	–	–
K8	0.594	–	–	A5	0.719	–	–	P15	0.675	–	–
K6	0.551	−0.343	–	A6	0.701	–	–	P10	0.644	–	–
K12	0.545	–	–	A10	0.537	–	–	P14	0.632	–	–
K9	0.543	–	–	A9	0.536	–	–	P11	0.625	–	–
K13	0.518	–	–	A8	0.526	–	–	P12	0.609	–	–
K11	0.437	–	–	A4	0.443	–	−0.422	P9	0.609	–	–
K14	0.404	−0.379	–	A13	0.441	–	0.441	P16	0.468	–	–
K7	–	0.810	–	A2	–	0.907	–	P5	0.407	–	–
K10	–	0.803	–	A3	–	0.850	–	P7	–	0.830	–
K3	–	–	0.744	A1	–	0.679	–	P8	–	0.732	–
K1	–	–	0.723	A11	–	–	−0.770	P18	–	0.638	–
K2	–	–	0.452	A12	–	–	−0.618	P6	–	0.588	–
K5	–	–	0.407	–	–	–	–	P17	–	0.422	–
K4	0.350	–	0.371	–	–	–	–	P3	–	–	−0.806
K16	0.314	–	0.343	–	–	–	–	P2	–	–	−0.772
–	–	–	–	–	–	–	–	P4	–	–	−0.691
–	–	–	–	–	–	–	–	P1	0.401	–	−0.552

*Note*: Items with factor load <0.3 were omitted.

### 3.2 Knowledge

#### 3.2.1 Ways of transmission and prevention

There were 10 items (1, 2, 8, 9, 10, 11, 12, 13, 14 and 15). This factor was related to the cause of the disease, the ways of transmission and individual measures to prevent the disease transmission. The internal consistency coefficient of this subscale was 0.50.

#### 3.2.2 High‐risk groups

There were three items (5, 6 and 7). This factor was related to groups at risk of contracting the disease. The internal consistency coefficient of this subscale was 0.46.

#### 3.2.3 Symptoms and treatment

There were three items (3, 4 and 16). This factor was related to the signs and symptoms of the disease and the available treatments. The internal consistency coefficient of this subscale was 0.20.

### 3.3 Attitude

#### 3.3.1 Hope

There were three items (1, 2 and 3). This factor indicated that people believed in the successful control of the disease and hoped that it would be eliminated by the measures taken by the government and adherence to personal precautions. The internal consistency coefficient of this subscale was 0.77.

#### 3.3.2 Fear

There were eight items (4, 5, 6, 7, 8, 9, 10 and 11). This factor indicated that people considered this disease a dangerous global crisis and were concerned about its individual, family and social consequences. The internal consistency coefficient of this subscale was 0.74.

#### 3.3.3 View of the vaccine

There were two items (12 and 13). This factor indicates that people have a favourable view of the vaccine's effectiveness in disease control. The internal consistency coefficient of this subscale was 0.1.

### 3.4 Practice

#### 3.4.1 Adhering to personal and public hygiene

There were eight items (5, 9, 10, 11, 12, 13, 14 and 18). This factor indicates that personal hygiene, such as hand washing, respiratory hygiene, etc., should be observed to prevent infection. The internal consistency coefficient of this subscale was 0.78.

#### 3.4.2 Limiting their presence in society

There were five items (6, 7, 8, 15 and 17). This factor indicated that people limited their social presence by doing things such as being in the community only when necessary, not using public transportation, etc. The internal consistency coefficient of this subscale was 0.68.

#### 3.4.3 Protecting oneself in the community

There were five items (1, 2, 3, 4 and 16). This factor indicated that people protected themselves from COVID‐19 in public by taking measures such as wearing a mask and observing social distancing. The internal consistency coefficient of this subscale was 0.81.

The internal consistency values of the second stage for total knowledge and practices were 0.63 and 0.85, respectively. The frequency of unanswered items for knowledge, attitude and practice sections was from 0.1% to 0.8%, 1.2% to 1.5% and 1.4% to 2.6%, respectively, indicating the questionnaire's usability.

### 3.5 Scoring

The knowledge section, with 16 items, had three answers, including correct, incorrect and I do not know. Each true answer was scored 1, whereas each false or do not know answer received 0. The range of scores of ways of transmission and prevention, high‐risk groups, and symptoms and treatment subscales and total score would be 0–10, 0–3, 0–3 and 0–16, respectively. Higher scores indicated more knowledge. The attitude section had 13 items, the answers of which were based on a Likert scale with five options: strongly agree (5), agree (4), neutral (3), disagree (2) and strongly disagree (1). Thus, the range of fear, hope and view of the vaccine scores would be 8–40, 3–15 and 2–10, respectively. The score of subscales was reported instead of the total score. Higher scores in the subscales of fear, hope and view towards the vaccine indicated more fear, hope and a more positive view of the vaccine, respectively. The practice section had 18 items with yes or no answers. The score for each item ranged from 0 to 1. Therefore, the range of subscales including adherence to personal and public hygiene, limiting presence in society, and protect oneself in the community and total practice score would be 0–9, 0–5, 0–4 and 0–18, respectively. Higher scores indicated better practices.

The mean (SD) scores of knowledge; fear, hope and view of the vaccine; and practices were 13.93 (2.10), 23.61 (4.65), 11.32 (2.40), 6.19 (1.59) and 16.78 (2.54), respectively. According to the regression analysis, knowledge of ways of transmission and prevention; symptoms and treatment; and fear and hope were the related factors of practices by controlling the effect of other variables (Table 3). The recommended model explained 34.4% of the practice variances (*R* ^2^ = 0.35).

**TABLE 3:** Results of multiple linear regression for testing hypothesis.^a^

Variables	*B*	SE	Standard *β*	*t*	Sig.
(Constant)	6.983	1.078		6.476	0.000
Age	0.031	0.008	0.185	3.926	**0.000** ^b^
Gender					
Female	0.471	0.193	0.084	2.444	**0.015** ^b^
Male	–	–	–	–	–
Number of children	−0.152	0.068	−0.100	−2.237	**0.026** ^b^
History of COVID‐19 infection					
Yes	−0.223	0.193	−0.042	−1.153	0.249
No	–	–	–	–	–
History of COVID‐19 infection in family or friends					
Yes	0.366	0.201	0.070	1.821	0.069
No	–	–	–	–	–
Education					
Diploma and below	0.087	0.505	0.016	0.173	0.863
Undergraduate	−0.238	0.502	−0.040	−0.474	0.636
Master of sciences	0.304	0.557	0.031	0.547	0.585
PhD	–	–	–	–	–
The priority of the source of information about COVID‐19					
Nurses & doctors	−0.548	0.542	−0.039	−1.012	0.312
Family & friends	1.240	0.641	0.073	1.936	0.053
Newspaper	1.985	2.194	0.029	0.905	0.366
Social networks	−0.107	0.398	−0.014	−0.270	0.787
TV	0.218	0.352	0.037	0.621	0.535
Job					
Housewife	−0.521	0.493	−0.056	−1.057	0.291
Student	0.473	0.450	0.068	1.051	0.294
Employed	0.204	0.423	0.033	0.482	0.630
Unemployed	1.387	0.675	0.079	2.054	**0.040** ^b^
Not reported	−0.079	0.424	−0.015	−0.187	0.852
Retired	–	–	–	–	–
Marital status					
Single	−0.248	0.248	−0.041	−0.999	0.318
Married	–	–	–	–	–
Ways of transmission and prevention	0.227	0.072	0.120	3.171	**0.002** ^b^
High‐risk groups	0.177	0.155	0.045	1.145	0.252
Symptoms and treatment	2.425	0.234	0.351	10.358	**0.000** ^b^
Fear	0.043	0.018	0.078	2.343	**0.019** ^b^
Hope	0.160	0.036	0.147	4.463	**0.000** ^b^
View of the vaccine	0.051	0.055	0.031	0.925	0.355

^a^ Dependent variable: practices.

^b^ *p* < 0.05.

## 4 DISCUSSION

This study designed and validated the questionnaire of knowledge, attitude and practices towards COVID‐19 in a general population sample. The questionnaire had 47 items and three sections, including knowledge, attitudes and practices. The subscales of each section were determined. The questionnaire items were designed by reviewing the literature and considering the WHO, CDC and the Iranian Ministry of Health of Iran guidelines. A panel of experts (*n* = 10) examined the content validity of the questionnaire and gave their opinions. The internal consistency of the practice section was 0.85. Grove et al. (2012) stated that an internal consistency >0.7 is appropriate for newly designed instruments. The internal consistency of the total knowledge and view of the vaccine subscale of attitude was 0.63 and 0.1, respectively, which was less than acceptable. Considering that the items of this section were designed based on an extensive literature review and according to the experts' opinions, they remained in the questionnaire. However, some say that Cronbach's *α* coefficients should be between 0.3 and 0.7, and values higher than 0.7 indicate redundancy. Important information about the psychometric properties of a scale may be lost when scale developers and users rely only on *α* (Boyle et al., 2015).

As an advantage of this study over most previous studies, EFA was performed for construct validity to identify the dimensions of each part. Previous KAP questionnaires have been designed and validated for non‐medical undergraduate and nursing students (Park, 2021; Saefi et al., 2020). However, the present questionnaire was designed and validated for the general population. Many previous studies have examined the KAP of the general population, but they have not validated the questionnaires (Al‐Hanawi et al., 2020; Haque et al., 2020; Isah et al., 2020; Sakr et al., 2021; Xu et al., 2021; Zhong et al., 2020). However, questionnaire validation was another advantage of this study.

In addition to EFA, “hypothesis testing” was used according to COSMIN's guide to check the construct validity of the questionnaire (Mokkink et al., 2010). According to KAP (Fan et al., 2018), it was assumed that if the questionnaire really measures people's knowledge, attitude and practice, knowledge and attitude should be the determining factors of their practice. This hypothesis was confirmed in the present study for two subscales (hope and fear) of attitude and two subscales (ways of transmission and prevention and symptoms and treatment) of knowledge. KAP theory states that knowledge is the basis of behaviour alterations, and attitude is its driving force (Fan et al., 2018). The results of most of the previous studies are similar to the results of our study. Recent studies have shown a positive correlation between knowledge and attitude with practice (Afzal et al., 2021; Tomar et al., 2021; Yesuf & Abdu, 2022). In another study, poor knowledge about COVID‐19 was associated with poor practices (Akalu et al., 2020). According to some studies, people who had a more positive attitude took more actions to deal with COVID‐19 (Ferdous et al., 2020; Singh et al., 2022). When people have more awareness and knowledge of symptoms, causes of disease, and treatment and care needed, they are more likely to start preventive precautions and early treatment and care. In our study, those who had more hope had better practices. When people have a positive attitude towards the impact of preventive methods and global and national practices to control the pandemic, they may better accept and observe preventive precautions. On the other hand, sufficient knowledge of the effectiveness of preventive methods leads to positive attitudes and better practices (Akalu et al., 2020). Our study revealed that people with higher fear scores were better in practice. In a recent study, although participants had high scores of fear, most had good practices (Ali et al., 2023). The protection motivation theory assumes that the adoption of health or recommended protective behaviours is a direct function of the individuals' motivation to protect themselves. If people perceive that they are vulnerable to a serious health threat, a lot of fear will be created in them, increasing their motivation to perform preventive or protective behaviours (Floyd et al., 2000). Also, increasing awareness of the dangers caused by the COVID‐19 disease affects the motivation to change people's behaviour. In a study, the fear of contracting COVID‐19 affected people's environmental behaviours, including the pattern of water consumption, their use of disposable containers, traffic with personal vehicles, etc. (Salehi et al., 2022). Another study found that perceived threat severity and susceptibility could cause “travel fear,” which could lead to protective motivation and protective travel behaviours after the outbreak of the COVID‐19 pandemic (Zheng et al., 2021). In a recent study, perceived fear of COVID‐19 infection had a significant positive correlation with willingness to engage in preventive behaviours (Matlabi et al., 2022).

This study had some limitations. Since most people visiting health centres in Iran are women, 70% of the participants were female, making it difficult to generalize the results to the entire population. Besides, due to the unstable conditions of COVID‐19, the instrument's stability was not evaluated through test–retest reliability. The daily news about new symptoms, preventive measures and treatment was one of the reasons for the instability of the situation. However, one of the requirements for test–retest reliability is the stability of existing conditions (Mokkink et al., 2010). In addition, given the cross‐sectional nature of the study, causal relationships could not be assessed. Other confounding variables, such as income, may affect KAP, which were not investigated in this study. Considering that items of the practice section were answered with yes and no, the authors will examine the scale (frequency) of practices in future studies.

## 5 CONCLUSION

The 47‐item questionnaire developed in this study had acceptable validity and reliability. Thus, this questionnaire is helpful in nursing practice, research and policy. Nurses can use it to examine the general population's knowledge, attitudes and practices during the corona outbreak. In the assessment stage of the nursing process, nurses can collect data from their clients utilizing this questionnaire, plan and design their interventions accordingly, and evaluate the interventions' effects. Nursing researchers can use the questionnaire in quantitative and interventional research. For example, this questionnaire can identify areas that lack knowledge and then design educational content based on that. After implementing the interventions, the post‐test can be conducted with this questionnaire to check the effectiveness of the interventions. This questionnaire helps politicians to identify the existing shortcomings and make policies accordingly.

## AUTHOR CONTRIBUTIONS

All authors participated in study conception and design. ZK collected data. MV and HNA analysed data. MV and HNA wrote the manuscript. All authors read and approved the final manuscript.

## FUNDING INFORMATION

This study is financially supported by Tabriz University of Medical Sciences.

## CONFLICT OF INTEREST STATEMENT

The authors declare no conflicts of interest.

## ETHICS STATEMENT

The research was approved by the Ethics Committee of Tabriz University of Medical Sciences (ethical code: IR.TBZMED.REC.1399.985). Participants were informed regarding the research goals, voluntary participation, confidentiality and anonymity of data, and the right to leave the research at any desired time, and then, written informed consent was obtained.

## Data Availability

The data that support the findings of this study are available on request from the corresponding author. The data are not publicly available due to privacy or ethical restrictions.

## Knowledge

**Table:**

Items	Correct	Incorrect	I do not know
1. When infected people cough and sneeze, corona disease spreads through respiratory droplets.			
2. Corona disease can be transmitted by touching a surface or object with the virus and then touching the mouth, nose or eyes.			
3. The main clinical symptoms of corona disease are fever, fatigue, dry cough, body pain and shortness of breath.			
4. At present, there is no effective treatment for corona disease, but rapid symptomatic and supportive treatment can help patients recover more.			
5. Elderly people and those suffering from severe chronic diseases such as heart or lung diseases and diabetes are at risk of suffering from more severe complications of corona disease.			
6. Pregnant women are more susceptible to corona disease than non‐pregnant women.			
7. It is not necessary for children and teenagers to take precautions to prevent the transmission of corona disease.			
8. After being in a public place, after blowing, coughing or sneezing, people should wash their hands with soap and water or use a hand sanitizer containing at least 60% alcohol for at least 20 s.			
9. Ordinary people can wear the usual medical masks to prevent corona disease.			
10. People should only wear masks if infected with the virus or caring for someone suspected of having the corona disease.			
11. Healthy nutrition increases the body's immunity and resistance to corona disease.			
12. Isolation and treatment of corona patients effectively reduce the spread of the virus.			
13. To prevent the transmission of corona disease, people should avoid going to crowded places and using public transportation.			
14. Corona disease can be transmitted from pets to humans.			
15. The time between contracting the virus and the onset of symptoms is 1–14 days.			
16. Virus is the cause of this disease.			

## Attitudes

**Table:**

Items	Strongly agree	Agree	Neutral	Disagree	Strongly disagree
1. I believe that corona disease will be successfully controlled in the end.					
2. Our country's measures can help win against the corona disease.					
3. Compliance with the precautionary measures proposed by the Ministry of Health will prevent the spread of the coronavirus.					
4. Corona is a dangerous disease.					
5. There is a high possibility that I will get corona disease.					
6. Even if a person with corona infection recovers, some complications will plague him for the rest of his life.					
7. I fear I will die if I get corona disease.					
8. The outbreak of corona disease has increased my living expenses.					
9. Many lives have been destroyed due to the spread of the corona disease.					
10. The outbreak of this disease has caused emotional coldness between my family members and me.					
11. This disease is the most significant health challenge in the world.					
12. The corona vaccine will stop the disease.					
13. If needed, I volunteer in Corona's scientific research (such as tests of new drugs or new vaccines).					

## Practices

**Table:**

Items	Yes	No
1. I wear a mask if I have to be in crowded places and gatherings.		
2. I try to avoid going to crowded places.		
3. I avoid cultural behaviours such as shaking hands and kissing.		
4. I observe social distancing in my daily activities.		
5. I wash my hands with soap and water for at least 20 s after going to public places or blowing, coughing or sneezing.		
6. I only leave the house when necessary.		
7. I avoid eating food prepared outside the house.		
8. I do not use public transportation (taxi, bus, subway, plane, etc.).		
9. I avoid contact with people who are suspected of being sick.		
10. I was vaccinated.		
11. I go to the doctor if I have suspicious symptoms such as fever, shortness of breath and cough.		
12. I avoid touching my eyes, nose and mouth.		
13. When I cough or sneeze, I cover my mouth and nose with the crook of my elbow or a tissue paper.		
14. I throw the used tissue papers in the trash can with a lid.		
15. I do not use the swimming pool, sauna and jacuzzi during the corona outbreak.		
16. I try to use my credit card instead of cash in my purchases.		
17. I avoid contact with domestic and wild animals and birds.		
18. I disinfect my mobile phone or tablet several times a day.		
